# Akt kinase LANCL2 functions as a key driver in *EGFR*-mutant lung adenocarcinoma tumorigenesis

**DOI:** 10.1038/s41419-021-03439-8

**Published:** 2021-02-10

**Authors:** Yuqing Lou, Jianlin Xu, Yanwei Zhang, Wei Zhang, Xueyan Zhang, Ping Gu, Hua Zhong, Huimin Wang, Jun Lu, Baohui Han

**Affiliations:** Department of Respiratory Medicine, Shanghai Chest Hospital, Shanghai Jiao Tong University, Shanghai, China

**Keywords:** Cancer genomics, Non-small-cell lung cancer, Oncogenes, Translational research

## Abstract

Epidermal growth factor receptor (*EGFR*) is a key oncogene in lung adenocarcinoma (LUAD). Resistance to EGFR tyrosine kinase inhibitors is a major obstacle for *EGFR*-mutant LUAD patients. Our gene chip array, quantitative polymerase chain reaction validation, and shRNA-based high-content screening identified the Akt kinase lanthionine synthetase C-like protein 2 (LANCL2) as a pro-proliferative gene in the *EGFR*-mutant LUAD cell line PC9. Therefore, we investigated whether LANCL2 plays a role in promoting cell proliferation and drug resistance in *EGFR*-mutant LUAD. In silico clinical correlation analysis using the Cancer Genome Atlas Lung Adenocarcinoma dataset revealed a positive correlation between LANCL2 and EGFR expression and an inverse relationship between LANCL2 gain-of-function and survival in LUAD patients. The *EGFR*-mutant LUAD cell lines PC9 and HCC827 displayed higher LANCL2 expression than the non-*EGFR*-mutant cell line A549. In addition, LANCL2 was downregulated following gefitinib+pemetrexed combination therapy in PC9 cells. LANCL2 knockdown reduced proliferation and enhanced apoptosis in PC9, HCC827, and A549 cells in vitro and suppressed murine PC9 xenograft tumor growth in vivo. Notably, LANCL2 overexpression rescued these effects and promoted gefitinib + pemetrexed resistance in PC9 and HCC827 cells. Pathway analysis and co-immunoprecipitation followed by mass spectrometry of differentially-expressed genes in LANCL2 knockdown cells revealed enrichment of several cancer signaling pathways. In addition, Filamin A and glutathione S-transferase Mu 3 were identified as two novel protein interactors of LANCL2. In conclusion, LANCL2 promotes tumorigenic proliferation, suppresses apoptosis, and promotes gefitinib+pemetrexed resistance in *EGFR*-mutant LUAD cells. Based on the positive association between LANCL2, EGFR, and downstream Akt signaling, LANCL2 may be a promising new therapeutic target for *EGFR*-mutant LUAD.

## Introduction

Lung cancer is the most prominent cause of cancer mortality worldwide, with lung adenocarcinoma (LUAD) accounting for around 50% of all lung cancers^1^. Mutations within the epidermal growth factor receptor (*EGFR*) gene are a major cause of LUAD^2,3^. Clinical trial evidence suggests that *EGFR*-mutant LUAD patients can be effectively treated using first-line EGFR tyrosine kinase inhibitors (EGFR TKIs), such as gefitinib and erlotinib, in combination with the anti-folate chemotherapeutic agent pemetrexed^4^. Unfortunately, 20% of these patients either do no respond or acquire EGFR TKI resistance^5^. This could be due to the addition of secondary activating mutations within the *EGFR* gene, such as Thr790Met (T790M)^6^, as well as *MET* gene amplification^7^. However, many other causes of EGFR TKI resistance are currently uncharacterized^8,9^. Consequently, a deeper understanding of the molecular mechanisms underlying aberrant EGFR signaling and EGFR TKI resistance in *EGFR*-mutant LUAD cells is required to treat chemoresistant LUAD patients.

EGFR hyperactivates the serine/threonine protein kinase Akt^10–16^, which is known to play a key role in supporting cell survival, proliferation, and glucose metabolism^17^. Akt hyperactivity is a common hallmark of a variety of human cancers, including *EGFR*-mutant LUAD, making it an important therapeutic target for cancer treatment^18^. Akt activity is subject to post-translational regulation and researchers have identified more than 20 of these regulatory proteins over the past two decades, including inhibitors and activators of Akt activity^19^. Notably, the enzyme lanthionine synthetase C-like 2 (LANCL2) has been identified as a novel activator of Akt^20^. LANCL2 interacts with both inactive Akt and the Akt kinase mTOR Complex 2 to directly promote Akt phosphorylation and cell survival in response to mitogenic signals in human liver cells^20^. However, the role of LANCL2 in *EGFR*-mutant LUAD tumorigenesis and EGFR TKI resistance (if any) has not yet been evaluated.

Therefore, we determined the correlation between LANCL2 and EGFR, and whether LANCL2 gain-of-function is associated with poor survival in LUAD patients. Furthermore, we evaluated the role of LANCL2 in cell proliferation and drug resistance in *EGFR*-mutant LUAD using in vitro and shRNA-mediated LANCL2 knockdown studies. In vivo tumorigenic activity was measured using a well-established murine xenograft model. Our data will provide novel insights into *EGFR*-mutant LUAD cell proliferation.

## Methods

### Animal welfare statement

All animal procedures followed international, national, and institutional guidelines for humane animal treatment and complied with relevant legislation. Male BALB/c nude mice (age range: 8- to 10-weeks old; weight range: 17–22 g) were obtained from our institution’s Experimental Animal Center and were housed under specific pathogen free [SPF], room temperature conditions with a 12-h/12-h light/dark cycle (*n* = 3 mice per cage). Mice were provided standard rodent chow (Laboratory Rodent Diet 5058; PMI Nutrition International) and sterilized tap water ad libitum. Animal health and welfare was monitored by visual inspection of general appearance, behavior, food intake, fecal output, and body weight every three days. Isoflurane was employed as a procedural anesthetic. Mice were humanely sacrificed by cervical dislocation under isoflurane anesthesia. There was no mortality events outside of planned euthanasia.

### In silico analysis of the Cancer Genome Atlas-Lung Adenocarcinoma (TCGA-LUAD) data

Publicly-available data on the TCGA-LUAD cohort (*n* = 566) is retrievable from the GDC Data Portal of TCGA (https://portal.gdc.cancer.gov/). LANCL2 mRNA expression analysis, LANCL2-EGFR correlation analysis, and Kaplan–Meier survival analyses were performed on the whole TCGA-LUAD cohort using the cBioPortal for Cancer Genomics^21^ and the UALCAN portal^22^.

### Cell lines

The gefitinib-sensitive human *EGFR*-mutant LUAD cell lines PC9 (EGFR p.Glu746_Ala750del) and HCC827 (EGFR p.Glu746_Ala750del)^23,24^ as well as the non-*EGFR*-mutant LUAD cell line A549 were purchased from American Type Culture Collection. All cells were cultured in RPMI media (Thermo Fisher) and supplemented with fetal bovine serum (10%) and penicillin/streptomycin (100 U/ml) and cultured in a humidified environment at 37 °C with 5% CO_2_. Cell lines were tested negative for mycoplasma.

### Plasmid constructs and lentiviral delivery

The lentiviral GV115 plasmid (hU6-MCS-CMV-EGFP) used all shRNA-mediated knockout experiments was acquired from GeneChem (Shanghai, China). The RNAi Consortium provided the short hairpin RNA (shRNA) sequences against all target genes. An empty GV115 plasmid was used to construct the negative control shCtrl. In order to ensure efficient gene knockdown, three independent shRNA sequences were designed for each target gene and the three separate plasmids carrying the three unique shRNA sequences were mixed in equal proportion during GV115 lentiviral packaging. Cells were cultured until they reached 50% confluency and were then incubated with GV115 lentiviral particles and polybrene (4 μg/mL).

The lentiviral GV358 plasmids (Ubi-MCS-3FLAG-SV40-EGFP-IRES-puromycin) used for LANCL2 overexpression was also acquired from GeneChem. For LANCL2 overexpression, a synonymous *LANCL2* mutant was constructed in which the shLANCL2 binding sequence was mutated from GGAAGATCATTCATAATTT to GcAAaATtATcCAcAAcTT.

### Gene chip arrays

Two whole human cDNA gene chip arrays were performed here using a similar methodology: (i) untreated PC9 cells to determine the most abundant genes in PC9 cells, and (ii) shLANCL2-infected PC9 cells vs. shCtrl-infected PC9 cells to determine differentially-regulated genes arising from LANCL2 knockdown in PC9 cells.

Briefly, the RNeasy Plus Mini Kit (Qiagen) was used to extract RNA. RNA was transcribed into cDNA and the cDNA product was amplified using an OneArray plus RNA kit (Phalanx Biotech Group). The Human Whole Genome OneArray was incubated with Cy5-labeled RNA targets to allow for hybridization. The signal intensity was quantified via the Agilent Microarray Scanner (Agilent). Data transformation and normalization were performed with GeneSpring software. Standardization of the data was performed using the RMA algorithm.

For the shLANCL2 vs. shCtrl PC9 cell analysis, significant differences between groups were identified using a non-paired *t*-test corrected by the Benjamini–Hochberg method. A corrected *p* value (i.e., FDR) threshold of ≤ 0.05 and a fold-change threshold of greater than or equal to ±2.0 were applied to identify the differentially-regulated probes. The differentially-regulated genes were obtained therefrom using the priority rule of the probe set. The Heatmap.2 function from the gplots package in R Project was used for clustering analysis of the differentially-regulated genes. The Euclidean matrix distance calculation and complete linkage methods were employed to perform the clustering analysis.

In the untreated PC9 cell analysis, the top 32 most abundant genes were selected for quantitative RT-PCR (qPCR) validation. In the shLANCL2 vs. shCtrl PC9 cell analysis, all downstream genes identified by pathway analysis were selected for qPCR validation.

### Gefitinib + pemetrexed combination therapy

Where indicated, PC9 cells and HCC827 cells were subjected to 72-h exposure of PBS vehicle control or 10 nM gefitinib (IC_50_: 13 nM and 8 nM, respectively) plus 20 nM pemetrexed (IC_50_: 50 nM and 500 nM, respectively)^23^.

### Quantitative RT-PCR (qPCR)

TRIzol (Shanghai Pufel) was used to extract RNA, and the concentration and quality of total RNA were measured by Nanodrop spectrophotometer. Reverse transcription was performed according to the kit instructions (Promega M-MLV kit). The qPCR protocol was as follows: 95 °C for 30 s; 95 °C for 5 s and 60 °C for 30 s for a total of 45 cycles. The qPCR reaction reagent was SYBR Master Mixture (TAKARA, DRR041B0). The LANCL2 primers were (5′ to 3′): forward-GTG TAG CGA TGT GAT TTG GC and reverse-AAT GCT GGA AAC CGT GAT GT. The GAPDH primers were (5′ to 3′): forward-TGA CTT CAA CAG CGA CAC CCA and reverse-CAC CCT GTT GCT GTA GCC AAA. All other primers were designed and synthesized by Sheng Gong Co. Ltd. (Shanghai, China). GAPDH housekeeping gene expression was used to normalize target gene expression and generate 2^^-ΔΔCt^ data.

### High content screening (HCS) via lentiviral gene knockdown

Twenty-four hours after lentiviral infection (day 0), a HCS assay was performed in the infected PC9 cells by incubating them for 4 days (days 1–4) to assess cell proliferation. Cell counts were measured by fluorescence imaging microscopy every day. Quantitative results were analyzed with ArrayScan HCS software (Cellomics, Inc., Pittsburgh, PA, USA). To verify the results of the HCS, the top three gene targets, which produced the greatest suppression in PC9 cell proliferation, were selected for functional verification using individual shRNAs. qPCR was performed to verify efficient gene knockdown for each individual shRNA.

### CCK-8 assay

The CCK-8 assay was used to assess cell proliferation. In brief, 2000 cells per well were plated into a 96-well plate with 150 μL of media and incubated for 24 hours at 37 °C. Next, 10 μL of the CCK-8 solution (Sigma, 96992) was added for the final 4 h of the day prior to oscillation for 5 min. Absorbance values (450 nm, reference wavelength of 650 nm) were obtained on a microplate reader (M2009PR, Tecan Infinite). This was repeated for five days.

### Flow cytometric detection of cell apoptosis

Annexin V-APC (eBioscience, 88-8007) was used to evaluate cell apoptosis via flow cytometry. Annexin V monochromatic staining was used here since the lentiviral-infected cells were already labeled with GFP. Briefly, PC9 cells (5 × 10^5^) were plated in six-well plates. Trypsin (Gibco) was used to detach cells, then cells were washed with PBS for a total of three times before being resuspended in 1 × binding buffer (200 μl) and incubated with Annexin V-APC at room temperature for 15 min in the dark. After adjusting 1 × binding buffer volume, cells were analyzed on a BD FACS Aria II (BD Biosciences).

### Caspase-3/7 activity assay

The Caspase-Glo 3/7Assay Kit (Promega, G8091) was used to assay caspase-3/7 activity according to the kit instructions. Absorbance values (485 nm excitation, 520 nm emission) were obtained on a microplate reader (M2009PR, Tecan Infinite).

### Western blotting

Cells were lysed using pre-cooled 2 × Lysis Buffer (1 M Tris-HCl (pH 6.8), 2% 100 mM mercaptoethanol, 20% glycerin, and 4% SDS) for 15 min followed by ultrasound (200 W, four 5-s cycles, 2 s between cycles). The BCA protein assay was performed according to the kit instructions (Beyotime) to standardize each sample to a 2 μg/μL protein concentration. Lysates were then loaded, ran and separated on polyacrylamide gels. The protein was then transferred from the gel and onto PVDF membranes. Membranes were incubated at 4 °C overnight with the following primary antibodies (diluted in TBST solution with 5% skim milk as indicated): anti-LANCL2 (1:500, ab88860), anti-FLNA (1:1000, ab76289), anti-FASN (1:1000, ab128870), anti-TRIM25 (1:1000, ab167154), anti-YWHAB (1:1000, ab32560) (all from Abcam); anti-GSTM3 (1:1000, 15214-1-AP), anti- PKLR (1:1000, 22456-1-AP), anti-YWHAE (1:1000, 11648-2-AP) (all from Proteintech); and anti-GAPDH (1:2000, sc-32233, Santa Cruz Biotechnology). The following day, membranes were incubated for 1.5 h at room temperature with secondary horseradish peroxidase-conjugated goat anti-mouse IgG (1:5000, sc-2005, Santa Cruz Biotechnology) diluted in TBST solution with 5% skim milk. Finally, the Pierce ECL Western Blotting Substrate (Thermo) and autoradiography were used to detect and visualized proteins on the membrane and protein levels were quantified using the software Quantity One 4.6

### Nude murine xenograft model

The BALB/c nude mice (*n* = 30) were acclimatized for one week prior to PC9 cell injection. Animals were randomly segregated into the three experimental cohorts (*n* = 10 mice per cohort) using a random number generator. Lentivirus-infected PC9 cells (1 × 10^7^) diluted in 200 μl Matrigel (1:1 in culture medium, Corning Life Sciences) were subcutaneously injected into the right flanks of anesthetized nude mice. Sample size was determined based on previous studies using similar xenograft tumor models^25,26^. Xenograft tumors volumes were measured every week and monitored for a total of six weeks (the predetermined endpoint). Following humane sacrifice, the xenograft tumors were dissected out, weighed, and measured. Tumor volumes were calculated as follows: volume = 0.5 × length × width^2^.

### Co-immunoprecipitation/mass spectrometry (Co-IP/MS) analysis

For the Co-IP experiment, PC9 cells were infected with a 3 × FLAG-labeled LANCL2-overexpressing GV491 lentivirus or a negative control GV491 lentivirus. After puromycin selection, anti-FLAG M2 Affinity Gel Magnetic Beads (A2220, Sigma, St. Louis, MO, USA) were used to capture the fusion proteins according to the manufacturer’s instructions. The immunoprecipitates were washed with lysis buffer and boiled in sample loading buffer for immunoblotting analysis.

For the MS experiment, proteins were resolved on a 10% SDS-PAGE and stained with Coomassie Brilliant Blue (R250). The excised stained gel pieces were acetonitrile-dehydrated, vacuum-dried, and trypsin-digested. The trypsin-digested peptides were recovered, dried, and resuspended in 50% CAN and 0.1% TFA. The peptide mixture was subjected to nano-liquid chromatography-tandem MS (LC-MS/MS) with a LTQ Velos-Orbitrap MS (Thermo Scientific, Waltham, MA, USA) coupled to an Ultimate RSLC nano-LC system (Dionex).

### Pathway and functional enrichment analysis

We used ClueGO Cytoscape plugin^27^, Ingenuity Pathways Analysis (IPA, Ingenuity Systems, Inc., Redwood City, CA, www.ingenuity.com)^28^, and STRING v11^29^ bioinformatics tools to identify significantly enriched pathways, biological functions and disease, as well as functional relationships between genes and gene networks.

### Statistics

Data are reported as means ± standard deviations unless otherwise specified. All data was statistically analyzed using Student’s *t*-test (two-tailed) or one-way ANOVA with Bonferroni’s multiple comparison test. A *p* value of < 0.05 was deemed significant for all analyses.

## Results

### LANCL2 is a key gene contributing to PC9 cell proliferation

**Fig. 1:**
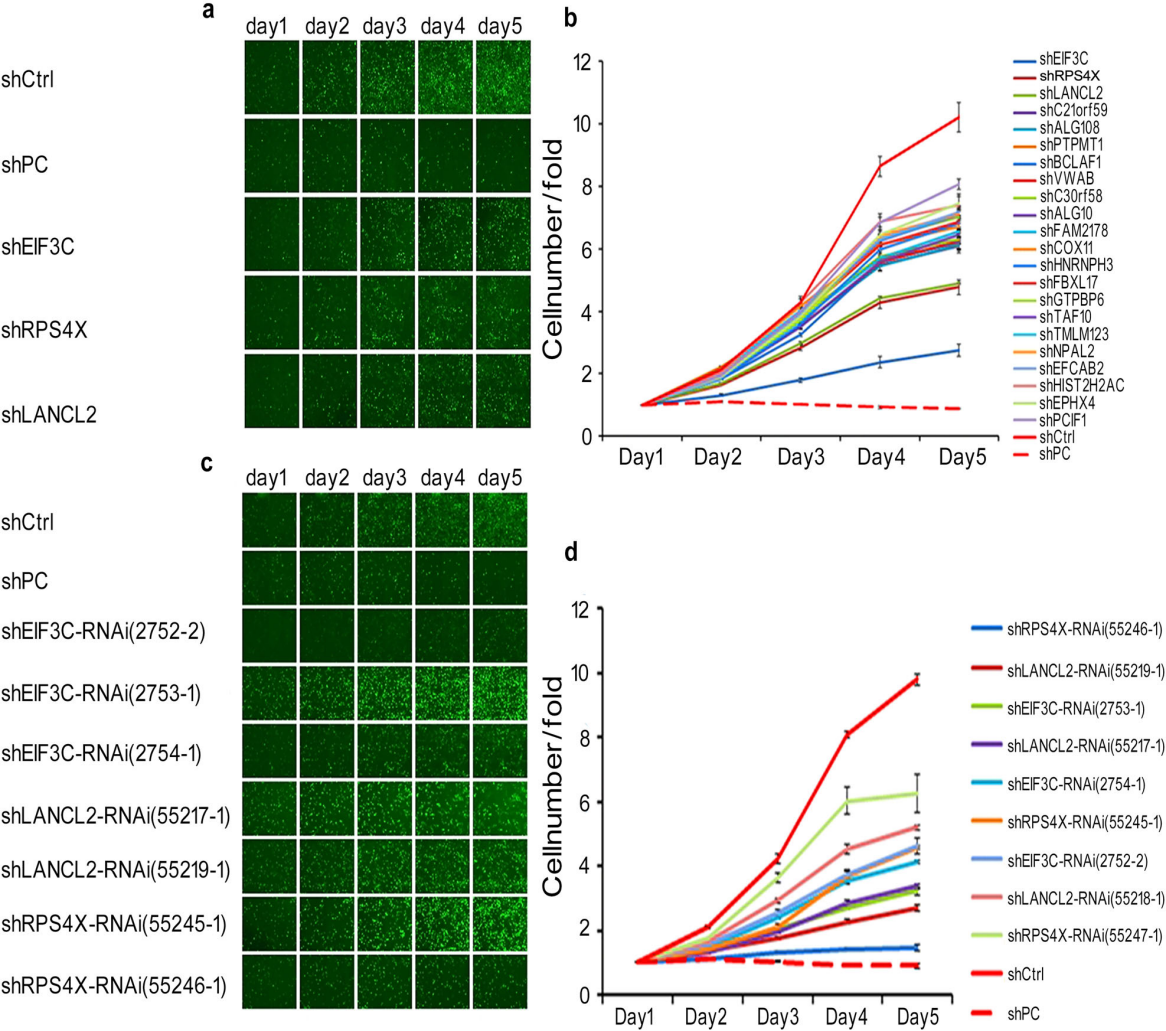
Identification of LANCL2 in PC9 cells using high content screening (HCS). **a** In the initial HCS on day 0, the candidate genes were individually silenced in PC9 cells using lentiviral shRNA-mediated knockdown. From day 1-4, HCS cell count analyses were conducted using the Cellomics ArrayScan. **b** Quantification of fold-changes in cell counts used as a measure of cell proliferation. **c** The shRNAs negating RPS4X, EIF3C, and LANCL2 expression were selected for individual verification in a secondary HCS. **d** Quantification of fold-changes in cell counts used as a measure of cell proliferation. Data presented as means ± SDs. All in vitro experiments: three biological replicates × three technical replicates.

We conducted a transcriptomic screen employing a gene chip array and follow-up qPCR validation to identify the most abundant gene transcripts in the PC9 LUAD cell line. qPCR analysis of the top 32 most-abundant gene transcripts identified from the gene chip array revealed several highly-abundant genes, including RPS4X, EIF3C, DHX9, TMEM123, and LANCL2 (Supplementary Fig. S1). Thereafter, we assessed the roles of these highly-abundant genes in PC9 cell proliferation using high content screening (HCS). On day 0, genes in PC9 cells were individually silenced using lentiviral shRNA-mediated knockdown, followed by cell count analysis conducted from day 1 to day 4. We found that certain shRNAs significantly suppressed PC9 cell proliferation over the four-day period (Fig. 1a and b). Specifically, the shRNAs targeting RPS4X, EIF3C, and LANCL2 displayed the most profound anti-proliferative effects; therefore, these shRNAs were selected for individual verification. After verification using HCS, we discovered that shLANCL2 more potently suppressed PC9 proliferation in vitro relative to shRPS4X or shEIF3C (Fig. 1c and d). This evidence suggested that LANCL2 plays a role in promoting PC9 cell proliferation; therefore, we selected LANCL2 as a target gene for further study.

### LANCL2 is positively correlated with EGFR in LUAD patients; high LANCL2 expression is associated with poor survival

We analyzed LANCL2 expression in the TCGA-LUAD cohort (*n* = 566) to ascertain the clinical relevance of LANCL2. No significant differences in LANCL2 mRNA expression were observed between LUAD tumors and normal lung tissue (Supplementary Fig. S2a). However, after screening the top 25 gene candidates with the highest expressional correlations with LANCL2, we identified the LUAD oncogene *EGFR* (Supplementary Fig. S2b). Notably, *EGFR* was the only one of these 25 candidate genes that possessed a significant mutational co-occurrence with *LANCL2* (Supplementary Fig. S2c). Moreover, LUAD tumor LANCL2 mRNA expression and LUAD tumor EGFR mRNA expression displayed a strong positive correlation (Pearson *r* = 0.63; Supplementary Fig. S2d). In addition, Kaplan–Meier survival analyses, using *LANCL2* mutational status (Supplementary Fig. S2e) and LANCL2 mRNA expression (Supplementary Fig. S2f), revealed that LANCL2 gain-of-function is significantly associated with inferior overall survival outcomes for LUAD patients. This combined evidence indicates that LANCL2 and EGFR expression are positively correlated, and high LANCL2 expression is associated with poor survival, in LUAD patients.

### LANCL2 knockdown suppresses *EGFR*-mutant LUAD cell proliferation

**Fig. 2:**
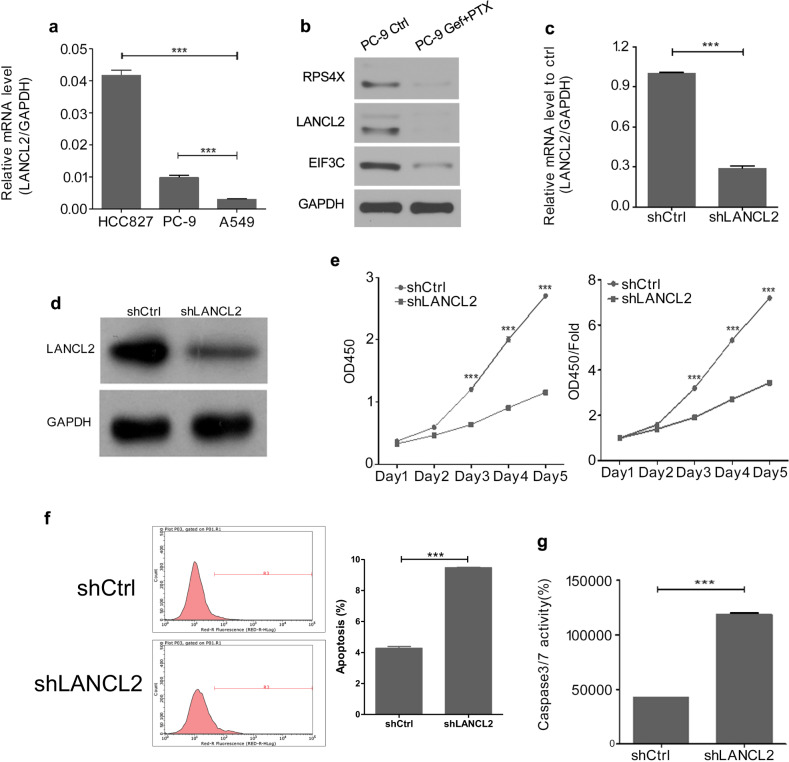
LANCL2 knockdown inhibits PC9 cell proliferation. **a** LANCL2 mRNA expression levels in PC9, HCC827, and A549 cells. **b** Immunoblotting analysis of RPS4X, LANCL2, and EIF3C protein expression in PC9 cells following vehicle control (Ctrl) or 10 nM gefitinib + 20 nM pemetrexed (Gef + PTX) combination therapy. **c** mRNA and **d** protein expression levels of LANCL2 in shLANCL2- or shCtrl-infected PC9 cells. **e** CCK-8 cell proliferation assay (raw OD450 data (left) and relative fold-change in OD450 (right)) and **f** flow cytometry analysis of apoptosis using Annexin V staining in shLANCL2- and shCtrl-infected PC9 cells. **g** Caspase-3/7 activity in shLANCL2- and shCtrl-infected PC9 cells. *p < 0.05; **p < 0.01; and ***p < 0.001 vs. shCtrl. Data presented as means ± SDs. All in vitro experiments: three biological replicates × three technical replicates.

We assayed three LUAD cell lines to determine their relative expression of LANCL2 and detected that the *EGFR*-mutant cell lines, namely PC9 and HCC827, displayed higher LANCL2 expression than the non-*EGFR*-mutant cell line A549 (Fig. 2a). These in vitro findings mirror the positive correlation observed between LANCL2 mRNA expression and EGFR mRNA expression in LUAD tumors (Supplementary Fig. S2). To assess the effects of gefitinib + pemetrexed combination therapy on the three key genes identified in the HCS (i.e., LANCL2, RPS4X, and EIF3C), we treated PC9 cells with vehicle control or 10 nM gefitinib + 20 nM pemetrexed (Gef + PTX). Immunoblotting analysis revealed that LANCL2, as well as RPS4X and EIF3C, were significantly downregulated following Gef + PTX combination therapy (Fig. 2b). This combined data suggest that LANCL2 may play a role in cell proliferation and drug resistance in *EGFR*-mutant LUAD cells.

**Fig. 3:**
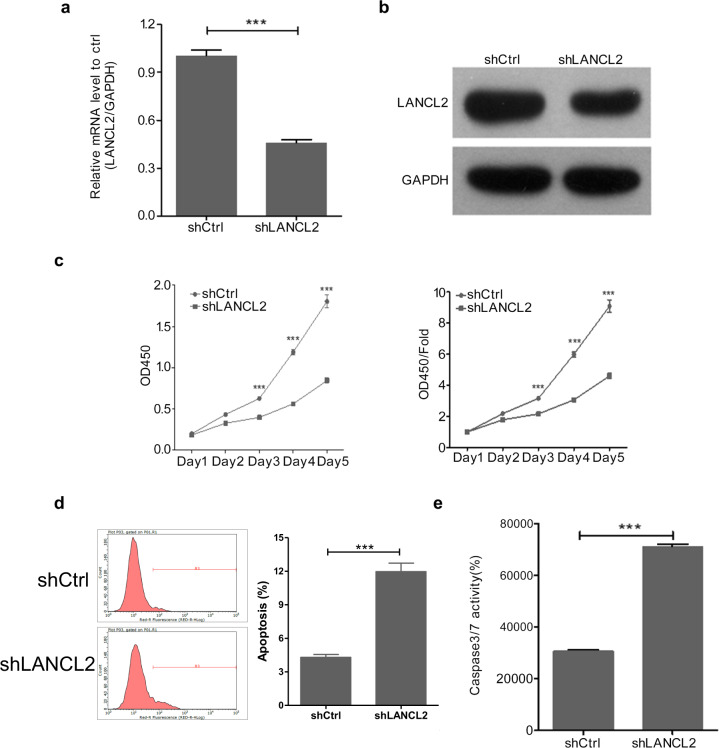
LANCL2 knockdown inhibits HCC827 cell proliferation. **a** mRNA and **b** protein expression levels of LANCL2 in shLANCL2- or shCtrl-infected HCC827 cells. **c** CCK-8 cell proliferation assay (raw OD450 data (left) and relative fold-change in OD450 (right)) and **d** flow cytometry analyses of apoptosis using Annexin V staining in shLANCL2- and shCtrl-infected HCC827 cells. **e** Caspase-3/7 activity in shLANCL2- and shCtrl-infected HCC827 cells. *p < 0.05; **p < 0.01; and ***p < 0.001 vs. shCtrl. Data presented as means ± SDs. All in vitro experiments: three biological replicates × three technical replicates.

To further investigate the role of LANCL2 in *EGFR*-mutant LUAD cells, PC9 cells were transfected with a shRNA against LANCL2 (55219-1, hereinafter referred to as shLANCL2) to knockdown LANCL2 expression. Both mRNA and protein expression levels of LANCL2 were notably reduced following shLANCL2 transfection (Fig. 2c and d). Additionally, the proliferation of shLANCL2-transfected PC9 cells was significantly lower than that of shCtrl-transfected PC9 cells (Fig. 2e). Furthermore, shLANCL2-transfected PC9 cells demonstrated elevated levels of cell apoptosis (Fig. 2f) and cleaved caspase-3/7 activity (Fig. 2g). To confirm these findings, we repeated the foregoing experiments in the *EGFR*-mutant HCC827 cell line. After effective LANCL2 knockdown (Fig. 3a and b), we evidenced similar findings in HCC827 cells with respect to cell proliferation (Fig. 3c), apoptosis (Fig. 3d), and caspase-3/7 activity (Fig. 3e).

To investigate the role of LANCL2 in non-*EGFR*-mutant LUAD cells, A549 cells were transfected with shLANCL2 to knockdown LANCL2 expression (Supplementary Fig. S3a and b). Similar to the *EGFR*-mutant LUAD cell lines, the proliferation of shLANCL2-transfected A549 cells was significantly lower than that of shCtrl-transfected A549 cells (Supplementary Fig. S3c). Furthermore, shLANCL2-transfected A549 cells demonstrated elevated levels of cell apoptosis (Supplementary Fig. S3d). These combined findings suggest that LANCL2 plays a pro-proliferative role in *EGFR*-mutant and non-*EGFR*-mutant LUAD cells.

### LANCL2 overexpression rescues LANCL2 knockdown-induced suppression of *EGFR*-mutant LUAD cell proliferation

**Fig. 4:**
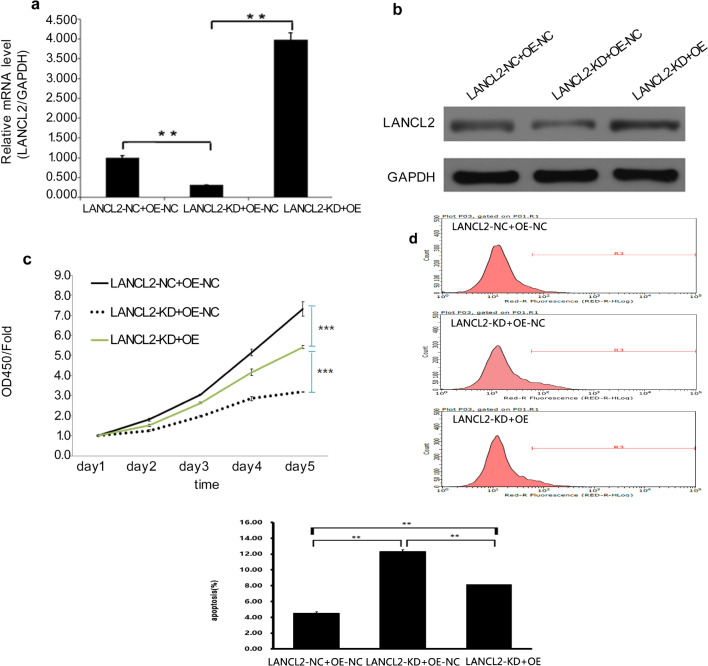
LANCL2 overexpression rescues effects of LANCL2 knockdown in PC9 cells. **a** mRNA and **b** protein expression levels of LANCL2 in negative control (LANCL2-NC + OE-NC), LANCL2 knockdown (LANCL2-KD + OE-NC), and LANCL2 knockdown with LANCL2 rescue overexpression (LANCL2-KD + OE) PC9 cells. **c** CCK-8 cell proliferation assay and **d** flow cytometry analyses of apoptosis using Annexin V staining in infected PC9 cells. *p < 0.05; **p < 0.01; and ***p < 0.001 vs. LANCL2-NC + OE-NC. Data presented as means ± SDs. All in vitro experiments: three biological replicates × three technical replicates.

**Fig. 5:**
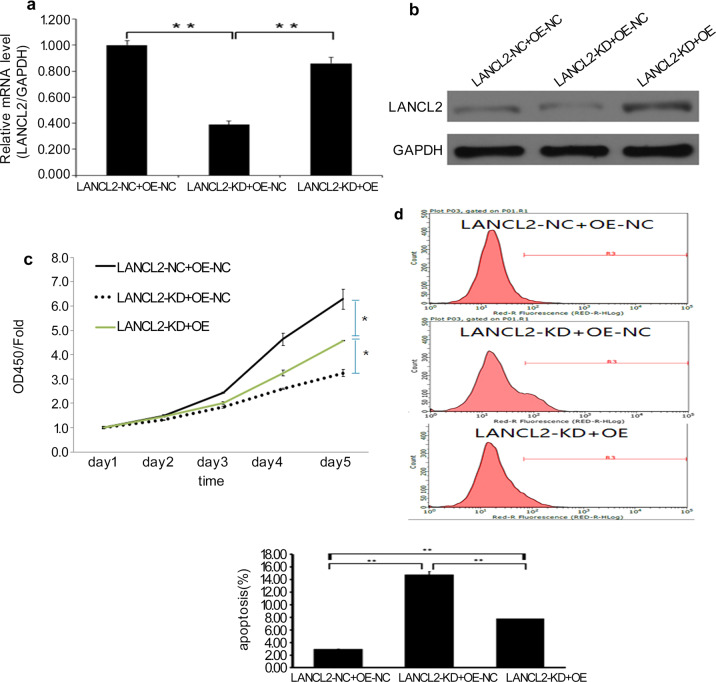
LANCL2 overexpression rescues effects of LANCL2 knockdown in HCC827 cells. **a** mRNA and **b** protein expression levels of LANCL2 in negative control (LANCL2-NC + OE-NC), LANCL2 knockdown (LANCL2-KD + OE-NC), and LANCL2 knockdown with LANCL2 rescue overexpression (LANCL2-KD + OE) HCC827 cells. **c** CCK-8 cell proliferation assay and **d** flow cytometry analyses of apoptosis using Annexin V staining in infected HCC827 cells. *p < 0.05; **p < 0.01; and ***p < 0.001 vs. LANCL2-NC + OE-NC. Data presented as means ± SDs. All in vitro experiments: three biological replicates × three technical replicates.

To validate the role of LANCL2 in PC9 and HCC827 cells, we devised a set of gene recovery experiments consisting of three experimental groups: a negative control group (LANCL2-NC + OE-NC), LANCL2 knockdown group (LANCL2-KD + OE-NC), and LANCL2 knockdown with LANCL2 rescue overexpression group (LANCL2-KD + OE). To effectively knockdown LANCL2 expression, LANCL2-KD + OE-NC cells were infected with a lentiviral vector containing shLANCL2. LANCL2-KD + OE cells, infected with a lentiviral vector containing a synonymous mutant of the *LANCL2* open reading frame that lacked the shLANCL2 binding site (Supplementary Fig. S4), led to effective LANCL2 overexpression in the presence of shLANCL2. Following infection, the knockdown and overexpression of LANCL2 in LANCL2-KD + OE-NC and LANCL2-KD + OE cells, respectively, was confirmed using qPCR and immunoblotting in PC9 cells (Fig. 4a and b). LANCL2-OE rescued the decreased cell proliferation produced by LANCL2-KD in PC9 cells (Fig. 4c). LANCL2-OE also rescued the increased apoptosis produced by LANCL2-KD in PC9 cells (Fig. 4d). To confirm these findings, we repeated the foregoing experiments in the HCC827 cell line. After effective LANCL2 knockdown and overexpression (Fig. 5a and b), we evidenced similar findings in HCC827 cells with regard to cell proliferation (Fig. 5c) and apoptosis (Fig. 5d).

### LANCL2 overexpression rescues LANCL2 knockdown-induced suppression of *EGFR*-mutant LUAD xenograft tumor growth

**Fig. 6:**
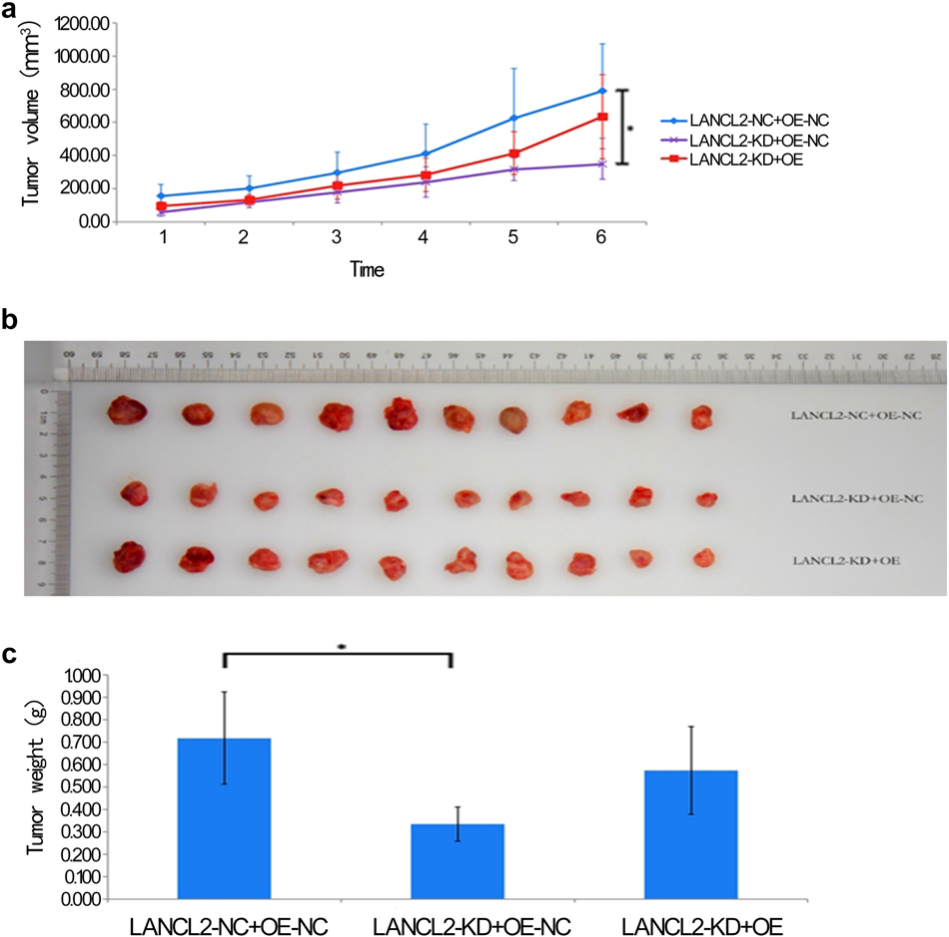
LANCL2 knockdown reduces PC9 xenograft tumor growth in vivo, while LANCL2 overexpression rescues effects of LANCL2 knockdown. **a** PC9 xenograft tumor volumes measured over a 6-week period (*n* = 10 mice per group). **b** Images of the extracted PC9 xenograft tumors after 6 weeks. **c** Quantification of the final PC9 xenograft tumor weights. *p < 0.05; **p < 0.01; and ***p < 0.001 vs. LANCL2-NC + OE-NC. Data presented as means ± SDs.

A nude murine PC9 xenograft tumor model was constructed to assess whether the loss or rescue of LANCL2 expression affects the growth of *EGFR*-mutant LUAD xenograft tumors in vivo. Therefore, 30 nude mice were injected with PC9 cells infected with either LANCL2-NC + OE-NC (*n* = 10 mice), LANCL2-KD + OE-NC (*n* = 10 mice), or LANCL2-KD + OE (*n* = 10 mice) as described above. Visible tumors were detectable 1-week post injection. We tracked tumor growth for 6 weeks and noted LANCL2-KD + OE-NC tumors grew slower than control LANCL2-NC + OE-NC tumors, while LANCL2-OE rescued this effect (Fig. 6a). At six weeks, the LANCL2-KD + OE-NC tumors were significantly smaller in size and weight than control LANCL2-NC + OE-NC tumors (Fig. 6a–c). LANCL2-OE rescued the decreased tumor size and weight produced by LANCL2-KD (Fig. 6a–c).

### LANCL2 overexpression promotes gefitinib + pemetrexed resistance in *EGFR*-mutant LUAD cells

To assess whether LANCL2 expression affects gefitinib+pemetrexed resistance in *EGFR*-mutant LUAD cells, we devised a set of LANCL2 overexpression experiments in treated PC9 and HCC827 cells using the following groups: a gefitinib-treated negative control group (Vector + Gefitinib), gefitinib+pemetrexed-treated negative control group (Vector + Gefitinib&Pemetrexed), and gefitinib + pemetrexed-treated LANCL2 overexpression group (LANCL2 + Gefitinib&Pemetrexed). Cells were treated with 10 nM gefitinib and 20 nM pemetrexed as indicated. Following infection, the overexpression of LANCL2 in PC9 cells was confirmed using qPCR and immunoblotting (Supplementary Fig. S5a and b). LANCL2 overexpression abrogated the suppressive effect of gefitinib+pemetrexed on PC9 cell proliferation (Supplementary Fig. S5c). In addition, LANCL2 overexpression nullified the positive effect of gefitinib+pemetrexed on PC9 cell apoptosis (Supplementary Fig. S5d) produced by LANCL2-KD. To confirm these findings, we repeated the foregoing experiments in the HCC827 cell line. After effective LANCL2 knockdown and overexpression (Supplementary Fig. S6a and b), similar findings were obtained with respect to cell proliferation (Supplementary Fig. S6c) and apoptosis (Supplementary Fig. S6d) in HCC827 cells.

### LANCL2 knockdown-induced DEGs associated with cancer-related signaling pathways and proteins

**Fig. 7:**
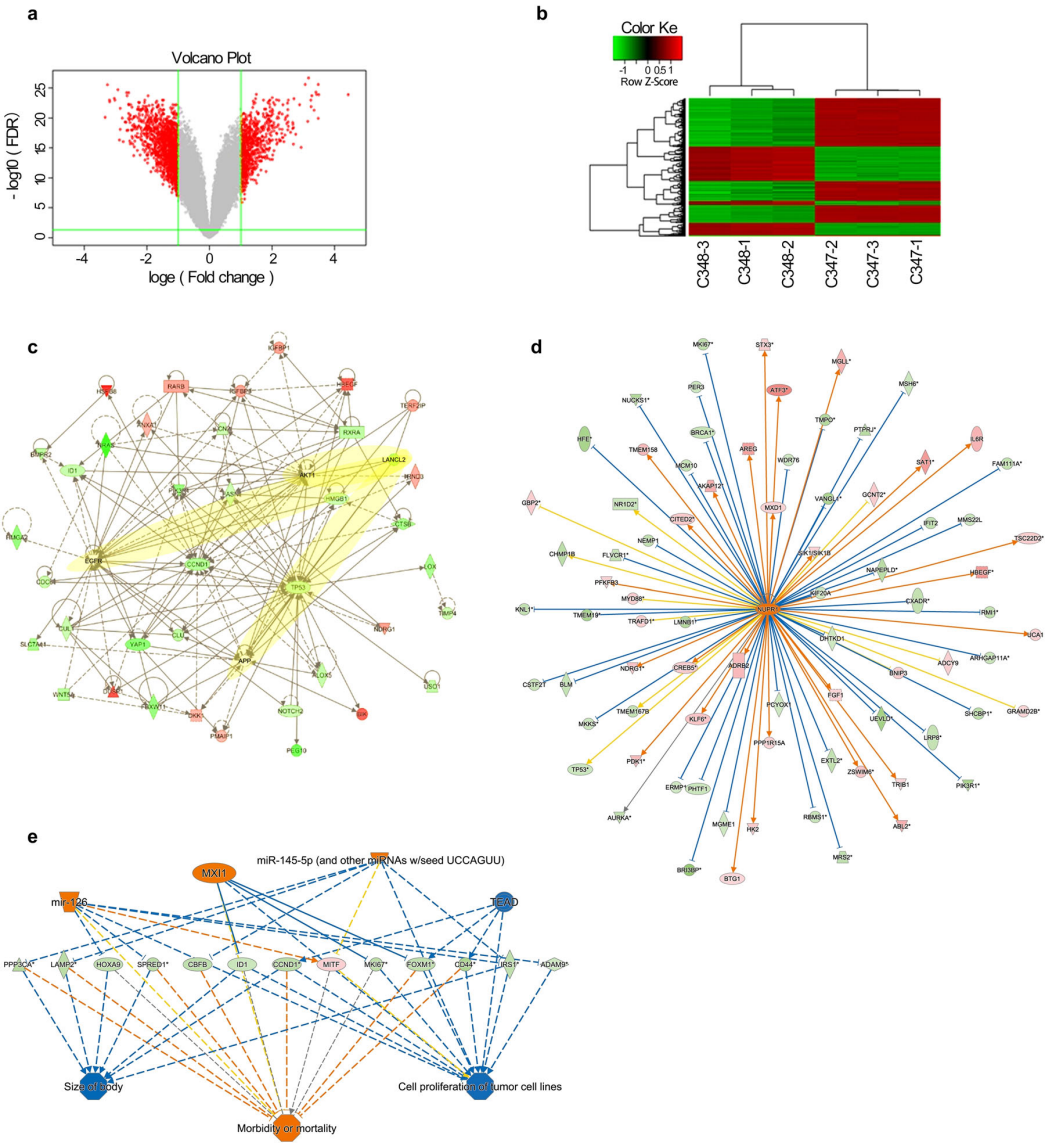
LANCL2 knockdown enriched cancer-related signaling pathways and proteins. **a** Volcano plot analysis of gene chip array results revealed 496 upregulated and 839 downregulated DEGs after LANCL2 knockdown (DEG criteria: *p* < 0.05; absolute fold-change >2.0). **b** Heatmap of DEGs after LANCL2 knockdown. Each row refers to one DEG. Each column represents one sample. The legend depicts the color code for Row Z-score fold-change values. **c** IPA interaction network analysis of DEGs detecting putative regulatory interactors of LANCL2. Note the highlighted putative interactions between LANCL2-EGFR, LANCL2-APP, and LANCL2-Akt. **d** IPA upstream regulator analysis of DEGs evidencing putative transcriptional regulators of LANCL2. **e** IPA downstream effects analysis of DEGs identifying putative dysregulated biological functions.

To improve understanding of the underlying molecular pathways responsible for the effects of LANCL2 on *EGFR*-mutant LUAD cells, we conducted gene chip array analysis in PC9 cells with and without LANCL2 knockdown. We discovered that the loss of LANCL2 produced 496 upregulated and 839 downregulated DEGs, based on our selected threshold values (Fig. 7a and b). Using these DEGs as inputs for ClueGO analyses identified pathways for protein localization (GO-BP), enzyme binding (GO-MF), and endoplasmic reticulum (GO-CC) were most enriched (Supplementary Fig. S7). Additionally, the ClueGO Kyoto Encyclopedia of Genes and Genomes (KEGG)/BioCarta pathway analyses of these DEGs revealed pathways in cancer, and the PPARA and Wnt signaling pathways were most significantly enriched (Supplementary Fig. S8). IPA was used for classical pathway analysis and revealed that DEGs were significantly enriched in HER2 breast cancer, non-small cell lung cancer, and melanoma signaling pathways (Supplementary Fig. S9a), while IPA disease and biological function analysis detected significant enrichment of cancer and organismal injuries and abnormalities (Supplementary Fig. S9b). We also performed an IPA interaction network analysis (which identifies regulatory events leading from signaling events to transcriptional effects; Fig. 7c), an IPA upstream regulator analysis (which predicts relevant transcriptional regulators and whether they are likely activated or inhibited; Fig. 7d), and an IPA downstream effects analysis (which identifies biological functions expected to increase or decrease; Fig. 7e). Notably, the IPA interaction network analysis revealed putative interactions between LANCL2 and EGFR, LANCL2 and amyloid precursor protein (APP), and LANCL2 and its traditional target Akt (Fig. 7c). We pursued qPCR validation of interactors of LANCL2 from the IPA interaction network analysis and found that loss of LANCL2 in PC9 cells resulted in the downregulation of several key genes (e.g., CCND1, WNT5A, and TP53) and upregulation of several key genes (e.g., APP, EGFR, and DKK1) (Supplementary Fig. S10a). Western blotting validated that LANCL2 knockdown significantly downregulated BMPR2 and NOTCH2 protein expression while significantly upregulating DUSP1 protein expression (Supplementary Fig. S10b).

### LANCL2 directly interacts with Filamin A (FLNA) and glutathione S-transferase Mu 3 (GSTM3)

To further explore mechanism of action of LANCL2 in *EGFR*-mutant LUAD cells, we screened for putative LANCL2 protein interactors in PC9 cells using Co-IP/MS analysis (Supplementary Fig. S11a). Seven proteins (FLNA, GSTM3, FASN, PKLR, TRIM25, YWHAB, and YWHAE) were identified as LANCL2 protein interactor candidates, based on the selection of Co-IP/MS proteins with an established relationship to cell proliferation (Supplementary Fig. S11b). Follow-up Co-IP immunoblotting demonstrated that LANCL2 interacts with FLNA and GSTM3 (Supplementary Fig. S11c). Notably, these two proteins have not been previously identified as LANCL2 protein interactors by STRING analysis (Supplementary Fig. S11d).

## Discussion

EGFR is a transmembrane tyrosine kinase receptor and key activator of intracellular Akt signaling via PI3K^9,30^. Akt hyperactivity has been evidenced to promote *EGFR*-mutant LUAD cell proliferation and chemoresistance^31^. LANCL2 plays a key role in promoting Akt hyperactivity in response to mitogenic signaling^20^; however, its involvement in EGFR TKI resistance in *EGFR*-mutant LUAD is largely uncharacterized. Herein, we evidenced that a positive correlation exists between LANCL2 and EGFR in LUAD patients, and LANCL2 gain-of-function is associated with poor survival in these patients. We showed that the *EGFR*-mutant LUAD cell lines PC9 and HCC827 displayed higher LANCL2 expression than the non-*EGFR*-mutant cell line A549. We also found that LANCL2 was downregulated following gefitinib+pemetrexed combination therapy in PC9 cells. Additionally, the shRNA-mediated knockdown of LANCL2 reduced proliferation and enhanced apoptosis in the *EGFR*-mutant LUAD cell lines PC9 and HCC827 in vitro and suppressed PC9 xenograft tumor growth in vivo. Moreover, we discovered that LANCL2 overexpression rescued these effects in LANCL2-silenced cells and promoted gefitinib+pemetrexed resistance in these *EGFR*-mutant LUAD cell lines. Furthermore, pathway analyses of DEGs from LANCL2 knockdown revealed significant enrichment for several cancer signaling pathways in PC9 cells. These combined results suggest that LANCL2 plays a key role in promoting *EGFR*-mutant LUAD.

In addition to its traditional tyrosine kinase function, EGFR signaling affects a number of tyrosine kinase-independent pathways^12,16,32^. Both tyrosine kinase-dependent and tyrosine kinase-independent EGFR signaling are able to hyperactivate pro-proliferative PI3K/Akt pathways^10^. Gefitinib-resistant cells do not respond to tyrosine kinase pathway inhibition, therefore targeting interactors in EGFR’s tyrosine kinase-independent pathways, such as LANCL2, to suppress Akt hyperactivity and cell proliferation could provide a useful strategy for bypassing EGFR TKI resistance in *EGFR*-mutant LUAD tumors. Interestingly, IPA analysis of the gene chip array data from LANCL2-knockdown PC9 cells evidenced that LANCL2 putatively interacts with EGFR as well as its traditional target Akt. Our follow-up qPCR analysis demonstrated that LANCL2 knockdown paradoxically promotes EGFR and APP mRNA expression levels in PC9 cells. APP is a putative protein interactor of EGFR^33^, and APP phosphorylation has been associated with poor survival in LUAD patients^34^. These findings may represent a regulatory-feedback mechanism to increase tumorigenic EGFR-Akt signaling in cells with LANCL2 knockdown-induced deficits in Akt activity. Therefore, this putative LANCL2-APP-EGFR-Akt regulatory sub-network should be further investigated.

Co-IP/MS analysis identified two novel protein interactors for LANCL2, namely FLNA and GSTM3, which may influence pro-proliferative effects of LANCL2 in *EGFR*-mutant LUAD cells. Filamin A (FLNA) is a cytoskeletal scaffolding protein that plays a key role in cellular signal transduction and is aberrantly expressed in several cancers^33^. Silencing FLNA expression in A549 *EGFR*-WT LUAD cells activates EGFR signaling as well as cell proliferation, migration, and invasiveness in vitro. This suggests that FLNA is a negative regulator of EGFR activation in LUAD cells^33^. We speculate that binding between LANCL2 and FLNA may serve to negatively regulate FLNA activity, thereby promoting EGFR-Akt activity in *EGFR*-mutant LUAD cells. GSTM3 is a member of the glutathione S-transferase family of enzymes and participates in a wide variety of cellular functions, including the inhibition of apoptosis and detoxification of xenobiotics^35^. The dysregulation of GSTM3 expression is exhibited in several human malignancies, including lung, colorectal, prostate, and triple-negative breast cancer^36–39^. Although early research suggested a weak association exists between certain GST gene mutations (e.g., *GSTM1*, *GSTM3*, *GSTP1*, and *GSTT1*) and risk of lung cancer^40,41^, more recent work has not evidenced any definitive links between GSTM3 polymorphisms and lung cancer risk^42,43^. However, molecular research in cervical cancer cells has revealed that GSTM3 upregulation is associated with promoting cancer cell survival, proliferation, tumor progression, and aberrant nuclear factor kappa B (NF-κB) and mitogen-activated protein kinase signaling^44^; therefore, further research is required to clarify whether GSTM3 plays a role in *EGFR*-mutant LUAD.

It should be noted that LANCL2’s effects on proliferation and apoptosis were not unique to the *EGFR*-mutant LUAD cell lines, as the non-*EGFR*-mutant LUAD cell line A549 was also significantly affected by LANCL2 knockdown. This observation may be attributable to the high endogenous EGFR activity displayed by A549 cells relative to other LUAD cell lines^45^. Moreover, as gefitinib+pemetrexed combination therapy downregulates LANCL2 expression in PC9 cells, it may also produce a similar effect in non-*EGFR*-mutant LUAD cells. Further research on a panel of non-*EGFR*-mutant LUAD cell lines with differing levels of endogenous EGFR activity and LANCL2 expression is needed to address these issues.

## Conclusion

LANCL2 promotes tumorigenic proliferation, suppresses apoptosis, and promotes gefitinib + pemetrexed resistance in *EGFR*-mutant LUAD cells. Based on the positive association between LANCL2, EGFR, and downstream Akt signaling, LANCL2 may be a promising new therapeutic target for *EGFR*-mutant LUAD.

## Supplementary information

Supplemental Figure Legends

Supplemental Figure 1

Supplemental Figure 2

Supplemental Figure 3

Supplemental Figure 4

Supplemental Figure 5

Supplemental Figure 6

Supplemental Figure 7

Supplemental Figure 8

Supplemental Figure 9

Supplemental Figure 10

Supplemental Figure 11

## Data Availability

The data that support the findings of this study are available from the corresponding author upon reasonable request.
