# Supplementary material for: Akt kinase LANCL2 functions as a key driver in EGFR-mutant lung adenocarcinoma tumorigenesis
Source: Cell Death Dis. 2021 Feb 10;12(2):170. doi: 10.1038/s41419-021-03439-8 (PMC7876134; doi:10.1038/s41419-021-03439-8)
Supplement: Supplementary file 1 — Supplemental Figure Legends [file 41419_2021_3439_MOESM1_ESM.docx]

**SUPPLEMENTAL FIGURES FOR**

**Akt kinase LANCL2 functions as a key driver in EGFR-mutant lung adenocarcinoma tumorigenesis**

**Supplemental Figure 1. qPCR verification of the gene chip array results in PC9 cells.** qPCR verification of the top 32 most-abundant gene transcripts identified from the gene chip array analysis in PC9 cells.

**Supplemental Figure 2. LANCL2 positively correlates with EGFR in LUAD patients; high LANCL2 expression associated with inferior survival.** All analyses were performed in the TCGA-LUAD cohort (n=566). (a) LANCL2 transcript expression in normal tissue and LUAD tumors. (b) Top 25 gene candidates with the highest expressional correlations to *LANCL2*. (c) *EGFR* and *TP53* show the strongest mutational co-occurrence with *LANCL2*. (d) Significant positive correlation between LUAD tumor LANCL2 mRNA expression and LUAD tumor EGFR mRNA expression (Pearson *r* = 0.63). (e, f) Kaplan-Meier survival analyses by (e) *LANCL2* mutational status and (f) LANCL2 mRNA expression (high: top 25^th^ percentile vs. low/medium: bottom 75^th^ percentile).

**Supplemental Figure 3. LANCL2 knockdown inhibits A549 cell proliferation.** (a) mRNA and (b) protein expression levels of LANCL2 in shLANCL2- or shCtrl-infected A549 cells. (c) CCK-8 cell proliferation assay (raw OD450 data (left) and relative fold-change in OD450 (right)) and (d) flow cytometry analyses of apoptosis using Annexin V staining in shLANCL2- and shCtrl-infected A549 cells. **p*<0.05; ***p*<0.01; and ****p*<0.001 vs. shCtrl. Data presented as means ± SDs. All *in vitro* experiments: 3 biological replicates × 3 technical replicates.

**Supplemental Figure 4**. **Sequence of synonymous *LANCL2* mutant used for LANCL2 overexpression in PC9 and HCC827 cells.** Mutations to the shLANCL2 binding sites, which prevent LANCL2 knockdown by shLANCL2, have been indicated.

**Supplemental Figure 5. LANCL2 overexpression promotes gefitinib+pemetrexed resistance in PC9 cells.** (a) mRNA and (b) protein levels of LANCL2 in vector- or LANCL2-OE-infected PC9 cells. (c) CCK-8 cell proliferation assay (raw OD450 data (top) and relative fold-change in OD450 (bottom)) and (d) flow cytometry analyses on apoptosis using Annexin V staining in infected PC9 cells. **p*<0.05, ***p*<0.01, and ****p*<0.001 vs. Vector. Data presented as means ± SDs. All *in vitro* experiments: 3 biological replicates × 3 technical replicates.

**Supplemental Figure 6. LANCL2 overexpression promotes gefitinib+pemetrexed resistance in HCC827 cells.** (a) mRNA and (b) protein levels of LANCL2 in vector- or LANCL2-OE-infected HCC827 cells. (c) CCK-8 cell proliferation assay (raw OD450 data (top) and relative fold-change in OD450 (bottom)) and (d) flow cytometry analyses on apoptosis using Annexin V staining in infected HCC827 cells. **p*<0.05, ***p*<0.01, and ****p*<0.001 vs. Vector. Data presented as means ± SDs. All *in vitro* experiments: 3 biological replicates × 3 technical replicates.

**Supplemental Figure 7. Results of the ClueGO Gene Ontology (GO) analysis from shLANCL2 versus shCtrl PC9 cells.**

**Supplemental Figure 8. Results of the ClueGO Kyoto Encyclopedia of Genes and Genomes (KEGG)/BioCarta pathway analysis from shLANCL2 versus shCtrl PC9 cells.**

**Supplemental Figure 9. Results of the Ingenuity Pathways Analysis (IPA) from shLANCL2 versus shCtrl PC9 cells.** Results from the (a) IPA pathway enrichment analysis and (b) IPA disease and functional analysis.

**Supplemental Figure 10. Validation of Ingenuity Pathways Analysis (IPA) interaction network analysis from shLANCL2 versus shCtrl PC9 cells.** (a) qPCR and (b) Western blotting validation of LANCL2’s interactors in the IPA interaction network analysis (see Figure 7c). **p*<0.05; ***p*<0.01; and ****p*<0.001 vs. Negative Control (NC).

**Supplemental Figure 11. Identification of FLNA and GSTM3 as LANCL2 interactors using co-IP/MS analysis.** (a) Lysates from PC9 cells infected with a FLAG-LANCL2 plasmid were evaluated by immunoprecipitation (IP) using an anti-FLAG antibody. Pull-down complexes were subjected to SDS-PAGE. The extracted IP-protein complexes were analyzed by LC-MS/MS. (b) Diagram of LANCL2-interacting candidate proteins identified by co-IP/MS. (c) Seven candidate proteins were selected for co-IP immunoblotting in PC9 cells; LANCL2 interacts with FLNA and GSTM3. (d) Known protein interactors of LANCL2 identified via STRING analysis under low-confidence (0.150).
