# Supplementary figures and images for: Akt kinase LANCL2 functions as a key driver in EGFR-mutant lung adenocarcinoma tumorigenesis
Source: Cell Death Dis. 2021 Feb 10;12(2):170. doi: 10.1038/s41419-021-03439-8 (PMC7876134; doi:10.1038/s41419-021-03439-8)

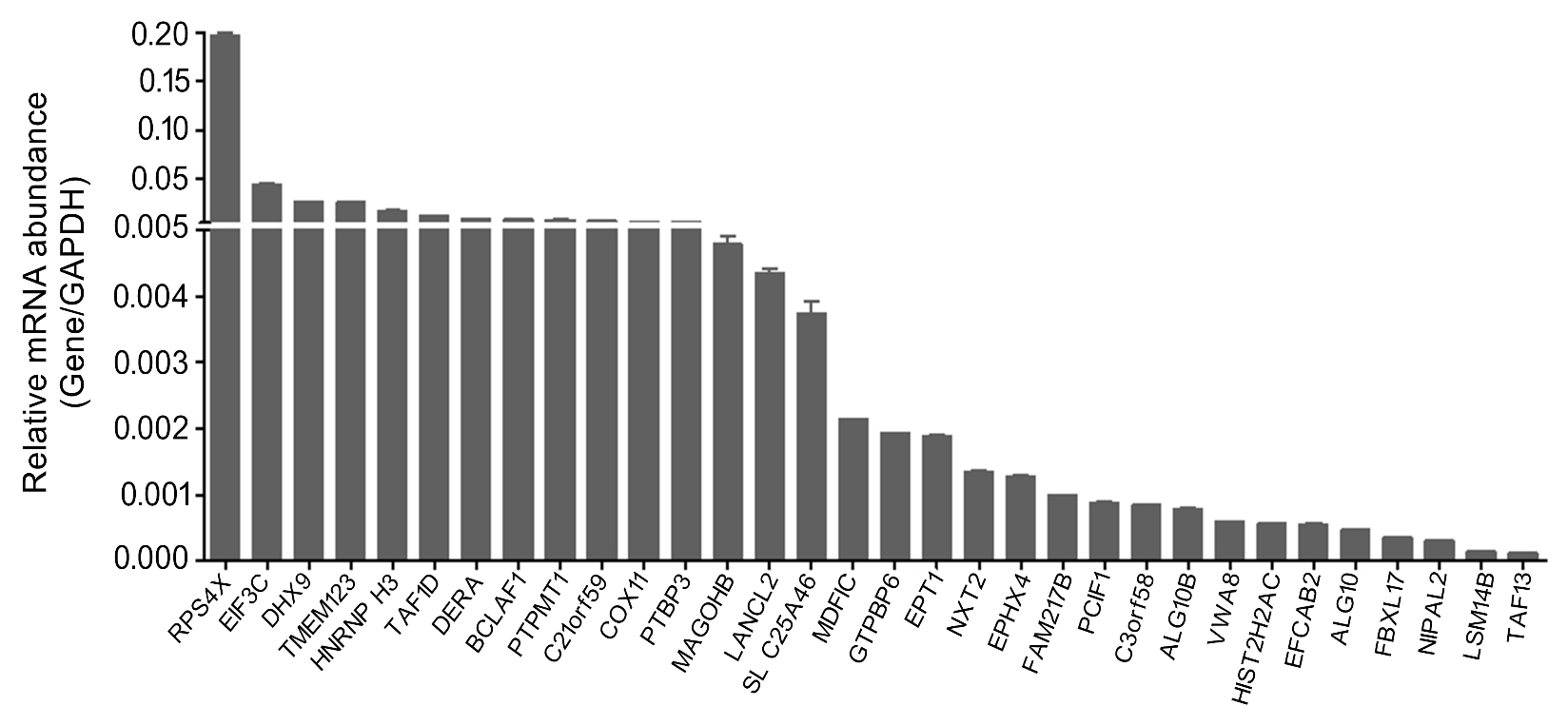

Supplement: Supplementary file 2 — Supplemental Figure 1 [file 41419_2021_3439_MOESM2_ESM.tif]

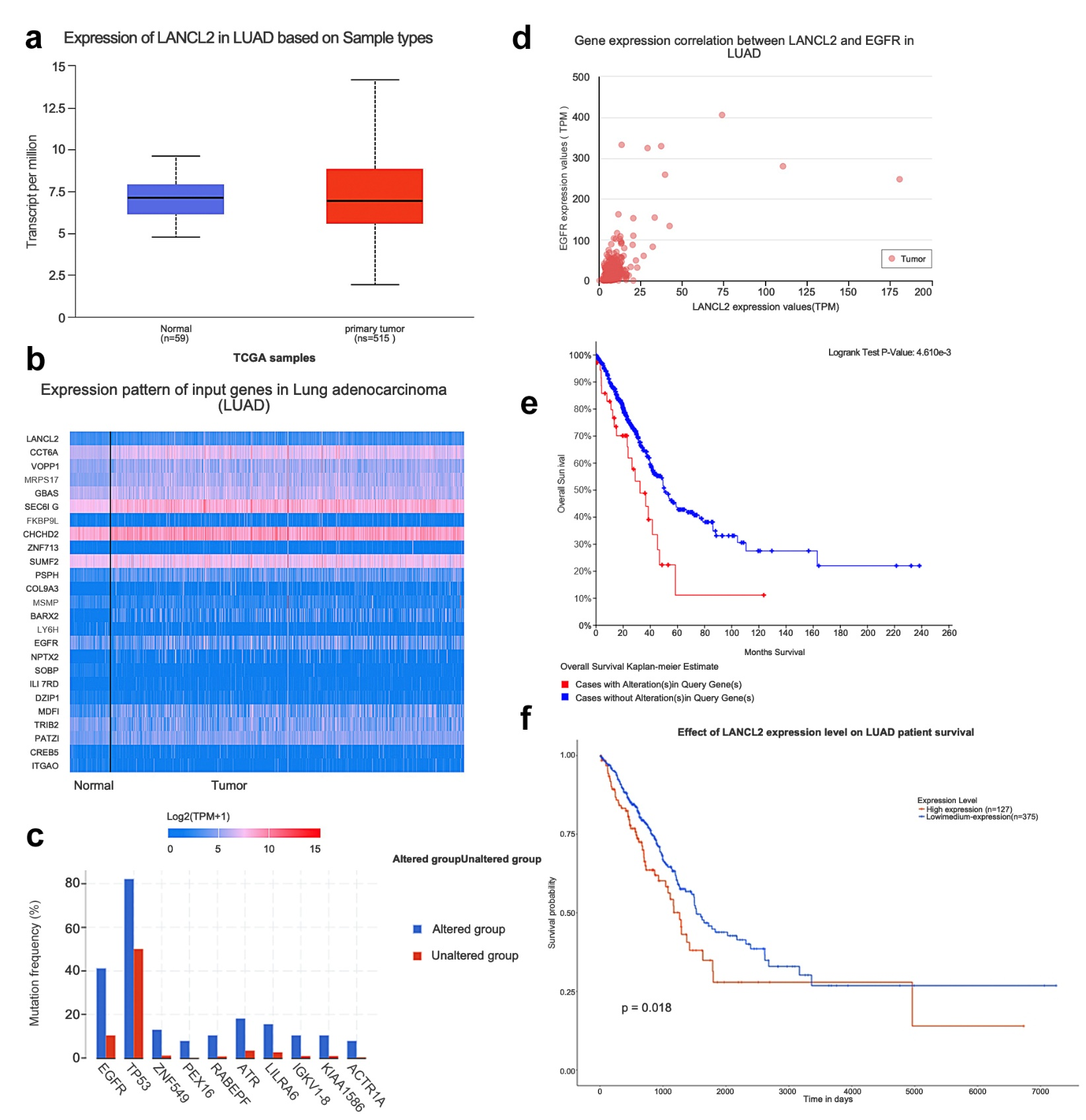

Supplement: Supplementary file 3 — Supplemental Figure 2 [file 41419_2021_3439_MOESM3_ESM.tif]

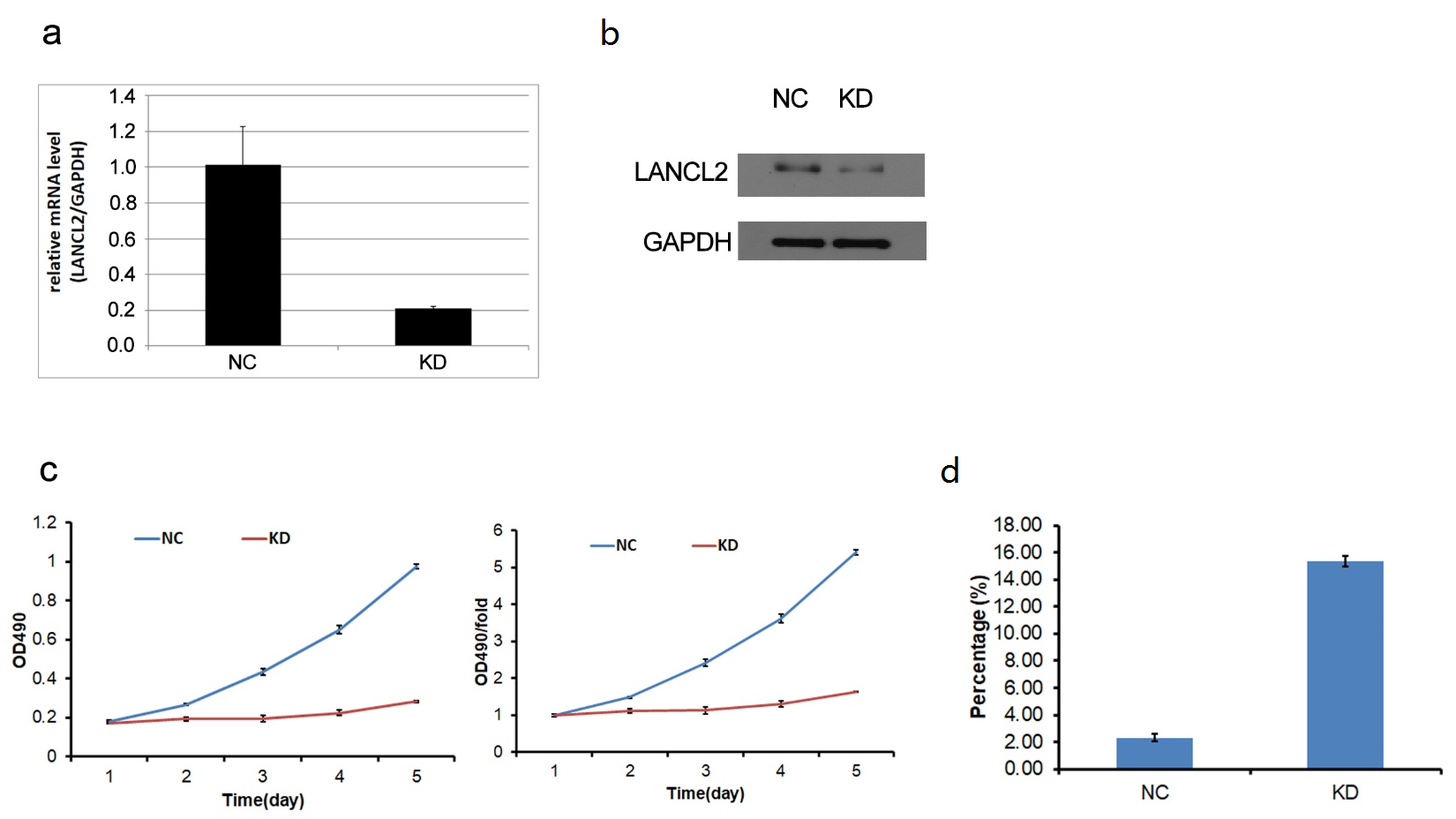

Supplement: Supplementary file 4 — Supplemental Figure 3 [file 41419_2021_3439_MOESM4_ESM.tif]

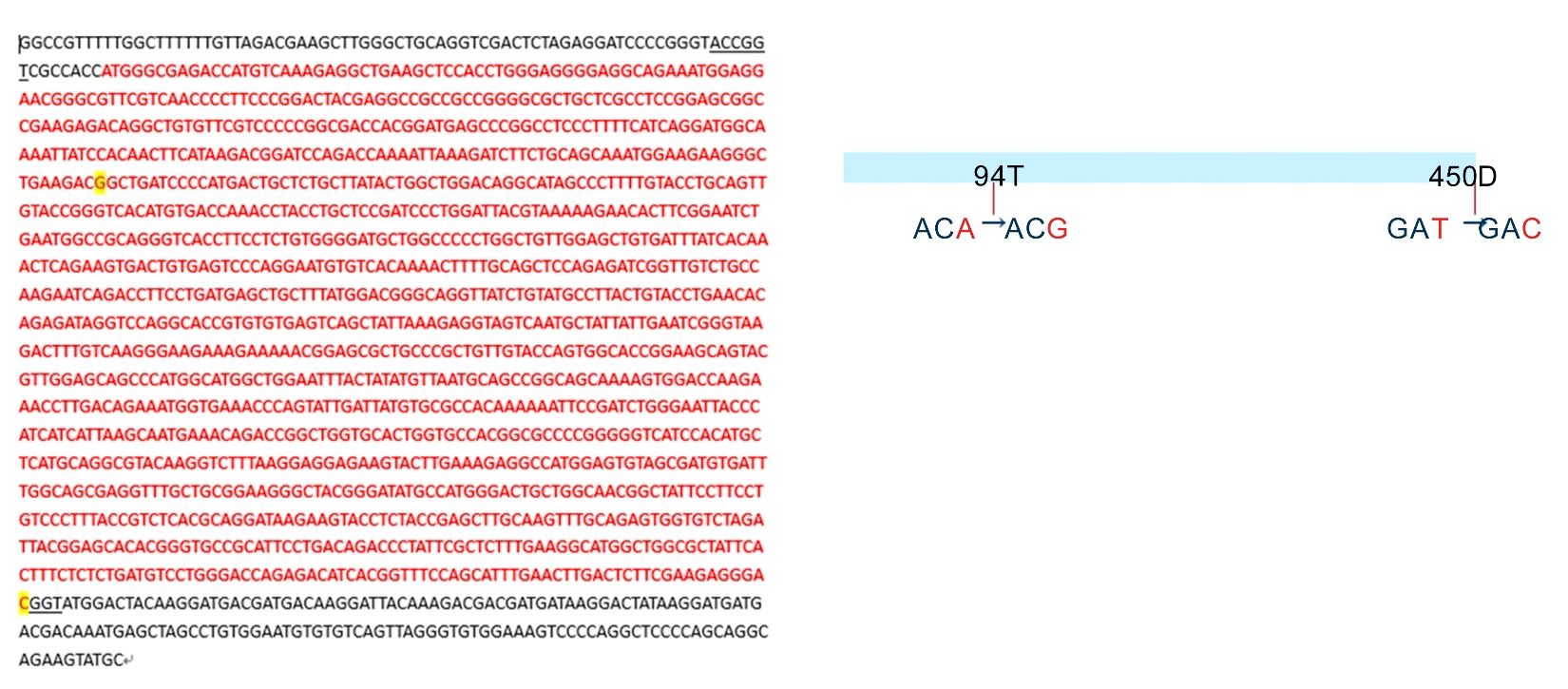

Supplement: Supplementary file 5 — Supplemental Figure 4 [file 41419_2021_3439_MOESM5_ESM.tif]

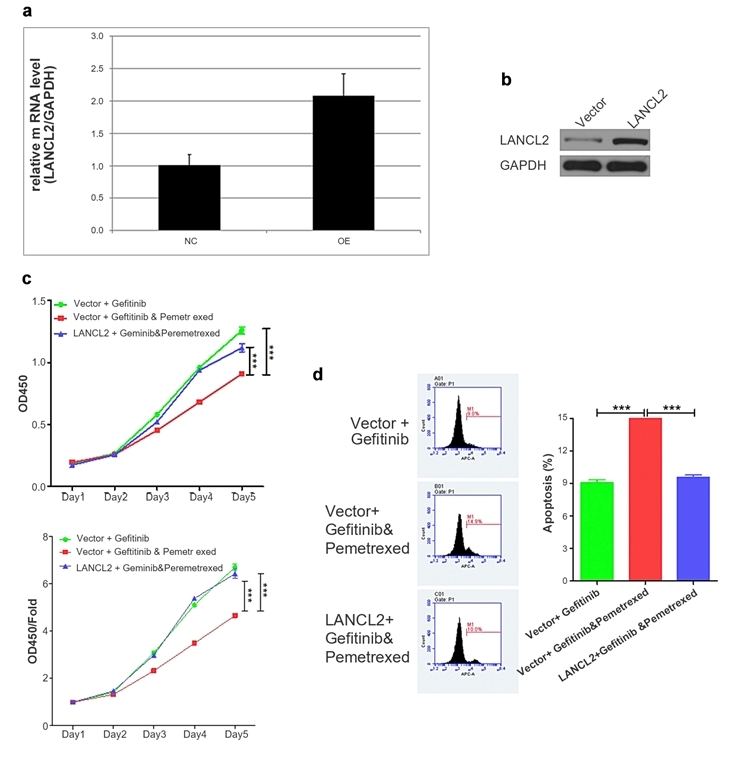

Supplement: Supplementary file 6 — Supplemental Figure 5 [file 41419_2021_3439_MOESM6_ESM.tif]

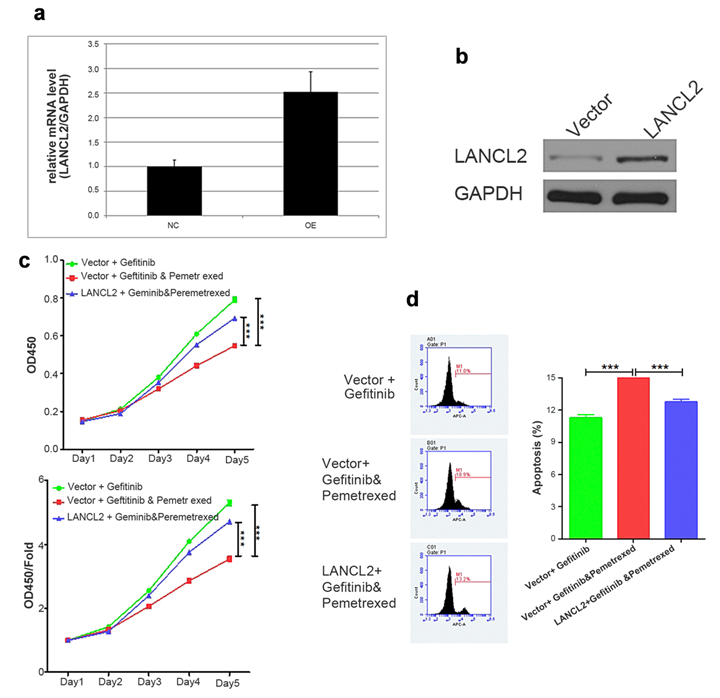

Supplement: Supplementary file 7 — Supplemental Figure 6 [file 41419_2021_3439_MOESM7_ESM.tif]

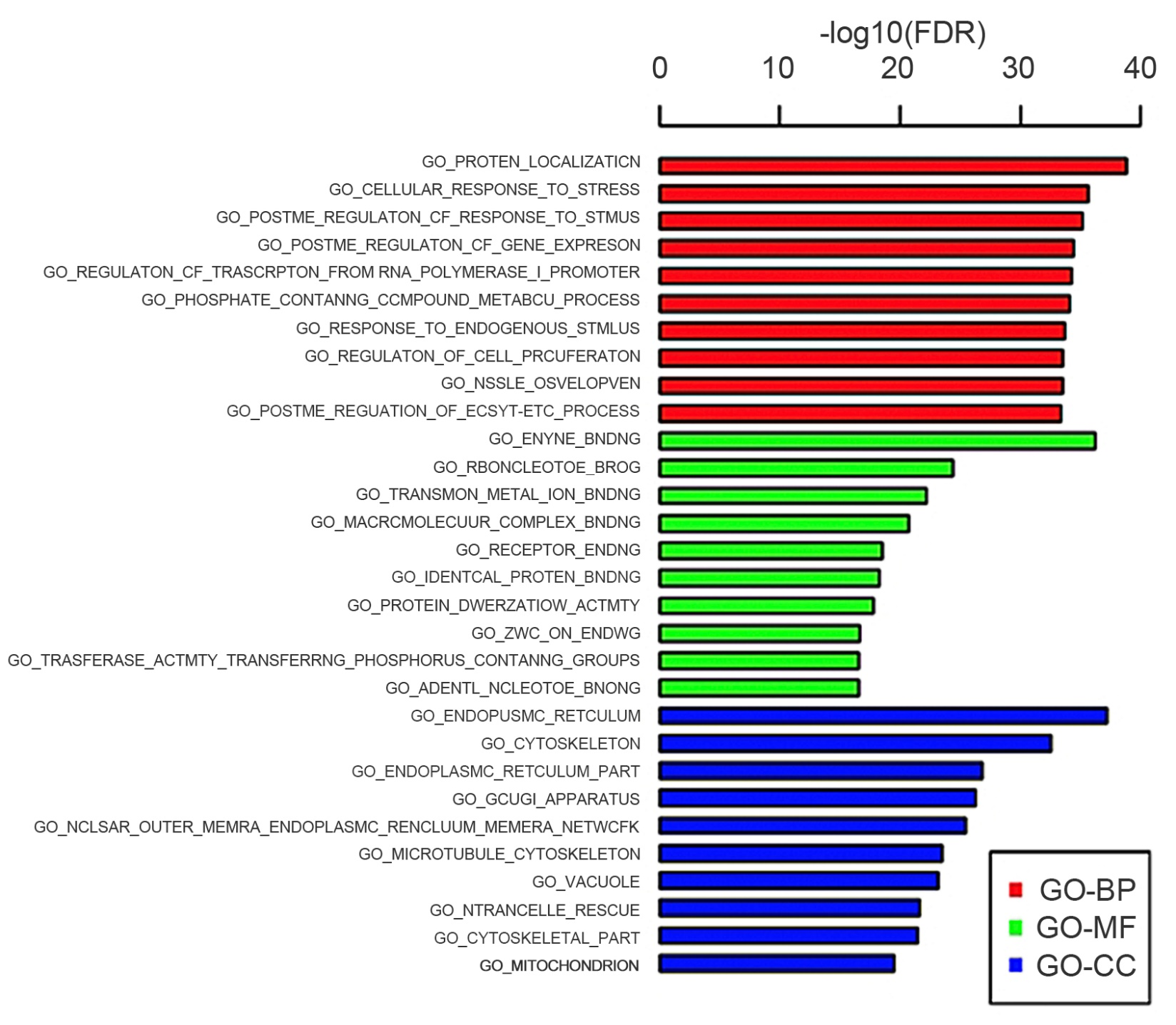

Supplement: Supplementary file 8 — Supplemental Figure 7 [file 41419_2021_3439_MOESM8_ESM.tif]

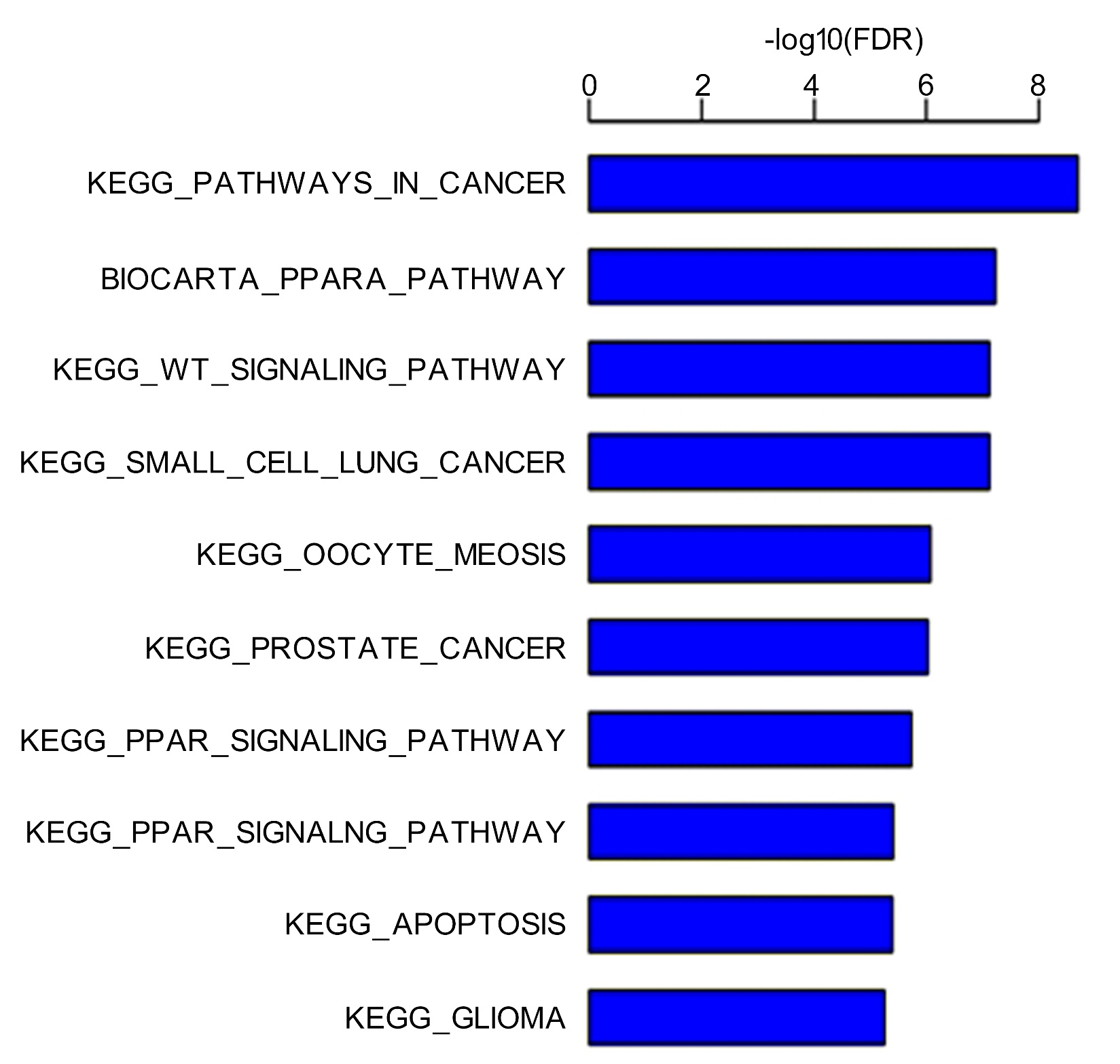

Supplement: Supplementary file 9 — Supplemental Figure 8 [file 41419_2021_3439_MOESM9_ESM.tif]

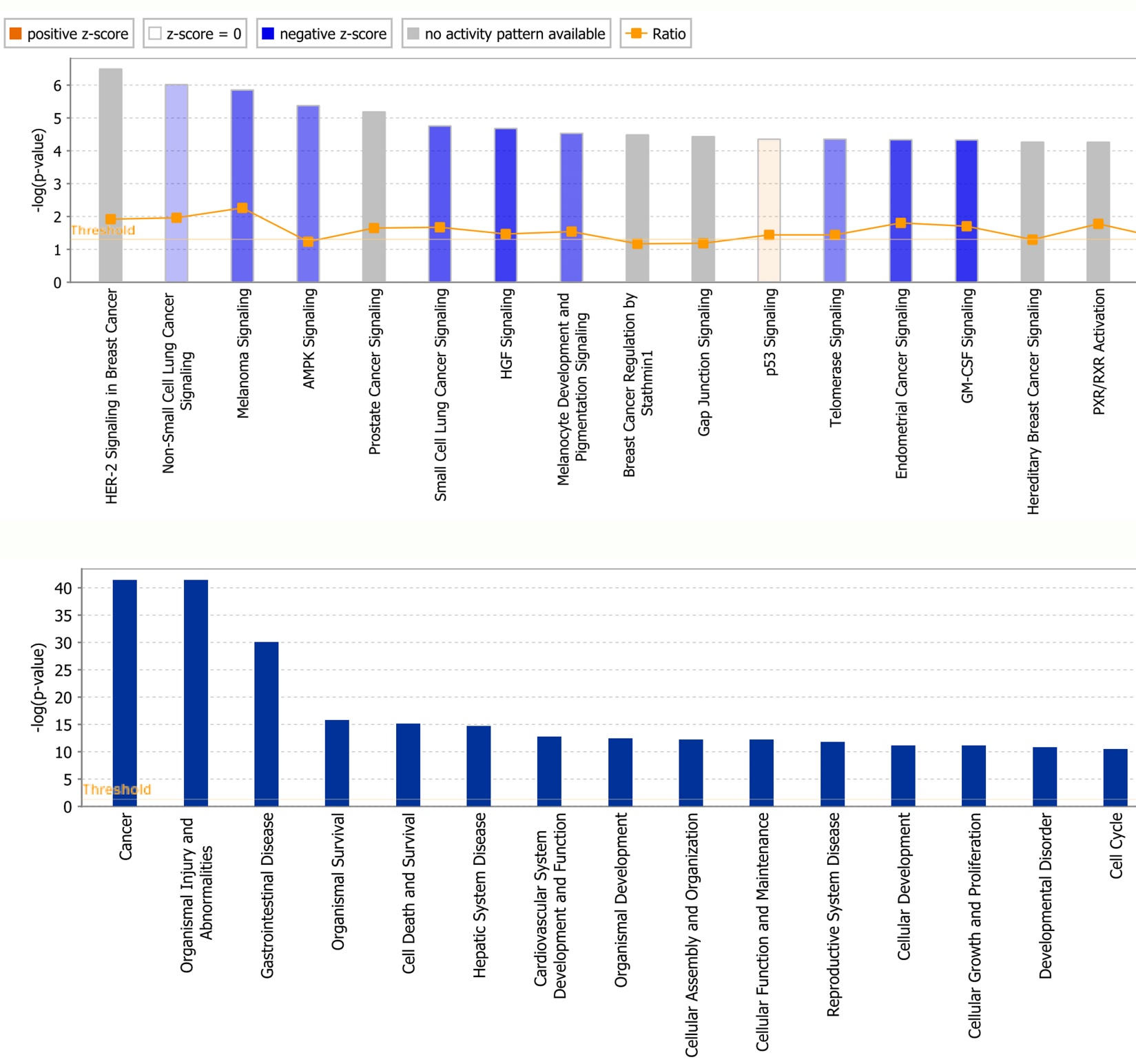

Supplement: Supplementary file 10 — Supplemental Figure 9 [file 41419_2021_3439_MOESM10_ESM.tif]

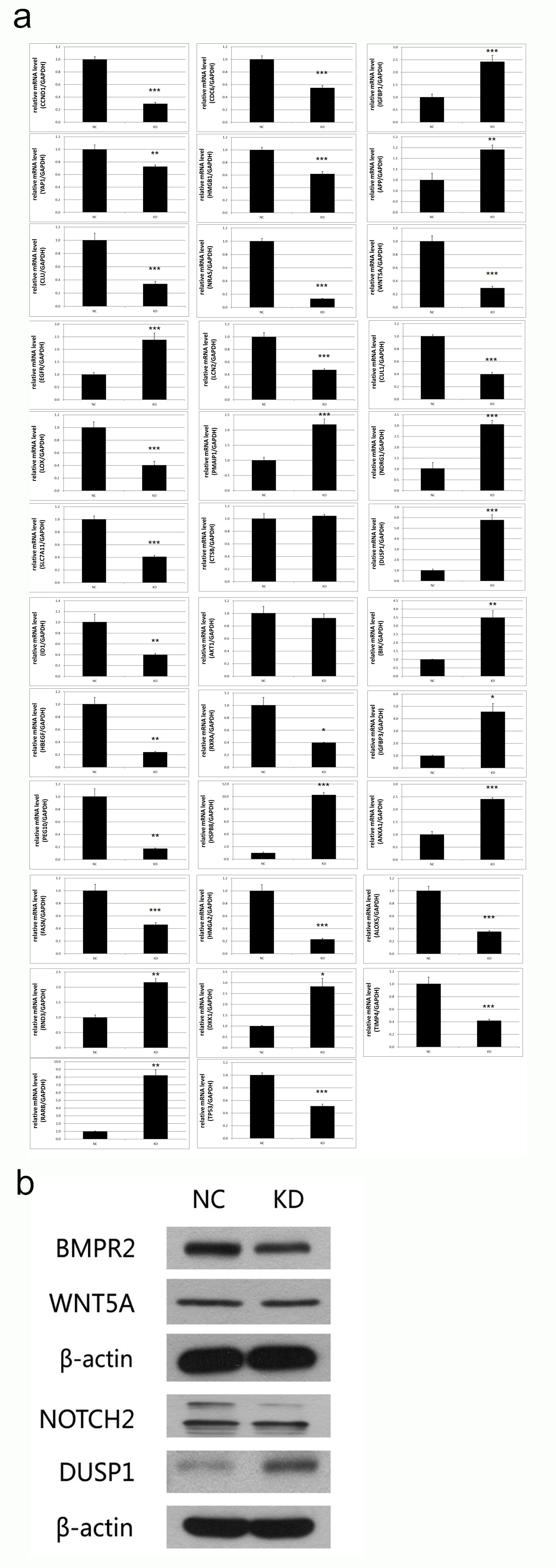

Supplement: Supplementary file 11 — Supplemental Figure 10 [file 41419_2021_3439_MOESM11_ESM.tif]

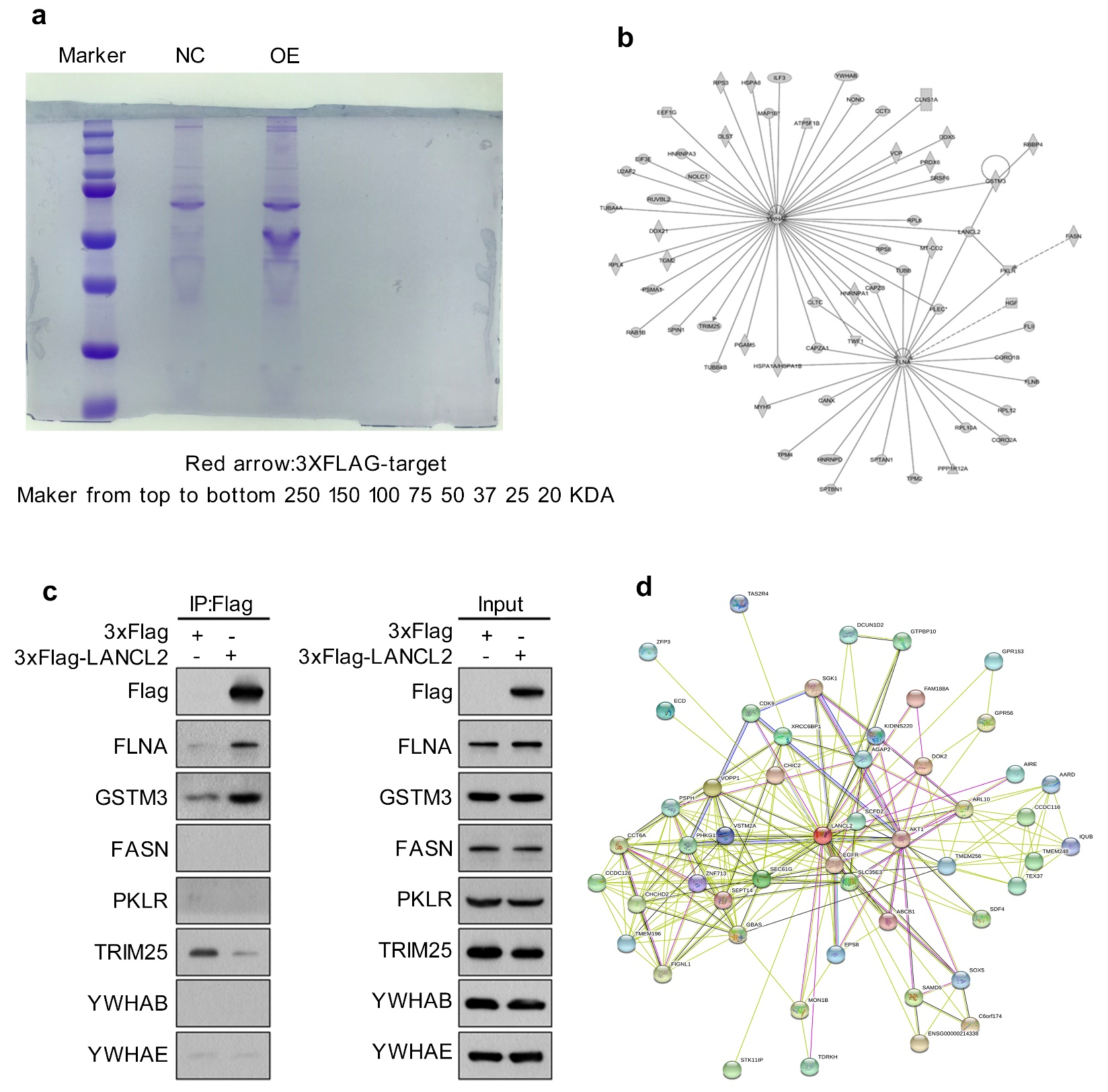

Supplement: Supplementary file 12 — Supplemental Figure 11 [file 41419_2021_3439_MOESM12_ESM.tif]
